# High-frequency ultrasound of the skin in systemic sclerosis: an exploratory study to examine correlation with disease activity and to define the minimally detectable difference

**DOI:** 10.1186/s13075-018-1686-9

**Published:** 2018-08-16

**Authors:** Hongyan Li, Daniel E. Furst, Hongtao Jin, Chao Sun, Xiaoping Wang, Lin Yang, Jingjing He, Yanru Wang, Aijing Liu

**Affiliations:** 1 Department of Immunology and Rheumatology, the Second Hospital of Hebei Medical University, Shijiazhuang, Hebei China; 2 Department of Rheumatology, Geffen School of Medicine, University of California, Los Angeles, CA USA; 3 The Affiliated Jiangning Hospital of Nanjing Medical University, Nanjing, Jiangsu China

**Keywords:** Systemic sclerosis, High frequency ultrasound, Disease activity, Skin thickness, Minimal detectable difference

## Abstract

**Background:**

Thickened skin is a major clinical feature in patients with systemic sclerosis (SSc). We investigated changes of skin thickness in patients with SSc using both high frequency ultrasound (HFU) and the modified Rodnan skin score (mRSS) to evaluate the feasibility of application of HFU in skin involvement and the relationship between HFU and clinical profiles.

**Methods:**

We recruited 31 consecutive patients with SSc and 31 age-matched and sex-matched healthy controls in this prospective, cross-sectional study. Skin thickness was measured by an 18-MHz ultrasonic probe at five different skin sites. Total skin thickness (TST) and skin thickness using categorical mRSS scores were recorded and compared to HFU. The European Scleroderma Trial and Research (EUSTAR) group Disease Activity Index (EUSTAR-DAI) and other clinical manifestations were assessed and analyzed.

**Results:**

TST in patients with SSc was thicker than in healthy controls (*P <* 0.001), and correlated positively with total mRSS and the EUSTAR-DAI and correlated negatively with disease duration (*P* < 0.05). Patients with higher TST had higher EUSTAR-DAI, mRSS, C-reactive protein (CRP) and lower diffusing capacity of the lung for carbon monoxide (DLCO) (*P* < 0.05). Even in patients who on clinical assessment were assigned an mRSS that suggested the skin thickness was normal. This was also true to mRSS locally of 1 and 2 (*P* < 0.01). The area under the receiver operator characteristic (ROC) curve was 0.831 and yielded sensitivity of 77.4% and specificity of 87.1% at the predicted probability of 7.4 mm as the optimal cutoff point to access skin thickness.

**Conclusions:**

In the study, HFU was able to measure skin thickness, it correlated quantitatively with a valid measure of SSc activity, and a minimal detectable difference was identified.

**Electronic supplementary material:**

The online version of this article (10.1186/s13075-018-1686-9) contains supplementary material, which is available to authorized users.

## Background

Systemic sclerosis (SSc) is an autoimmune disorder of unknown etiology, characterized by thickening and hardening of the skin and variable involvement of internal organs, with fibrosis and vasculopathy being the major pathological changes [1]. Skin tightening is the basic clinical hallmark for the diagnosis of SSc and is used to classify subtypes of the disease as well [2, 3].

Skin involvement in early diffuse SSc predicts the extent of visceral involvement, prognosis and mortality [4]. Improvement in the skin score (the semi-quantitative method used to assess skin thickening by palpation) indicates a favorable disease course and vice versa [5].

The modified Rodnan skin score (mRSS) was established by Rodnan in 1979 and is a validated method to evaluate skin thickening in SSc worldwide [6–8]. The mRSS is a popular parameter for reflecting changes in cutaneous involvement in patients with SSc because it is intuitive, comprehensive and repeatable in studies [8–10]. Improvement in the mRSS is associated with prolonged lifespan and with a good prognosis [5]. Nevertheless, the mRSS has some disadvantages such as lack of objectivity, bias among different examiners and failure to detect small degrees of skin change. Other techniques that are more sensitive, objective and reliable for assessment and evaluation are in development [11].

In recent years, high-frequency ultrasound (HFU) has frequently been applied beyond the musculoskeletal system. For example, HFU is used to detect skin thickness in patients with SSc because it can separate epidermis, dermis and subcutaneous layers [12, 13]. Moreover, it may allow early diagnosis of SSc skin involvement, because increasing skin thickness identified ultrasonically implies increasing disease severity [14–16]. However, HFU is subject to sampling artifacts and incompleteness, so its validity is disputed [17].

Using the validated European Scleroderma Trial and Research (EUSTAR) group Disease Activity Index (EUSTAR-DAI) in a cross-sectional analysis, with a large range of disease duration, we wished to compare HFU with the mRSS to further investigate skin thickness as a reflection of disease activity or severity, and to define a minimally detectable difference (MDD) by HFU.

## Methods

### Patients

We prospectively recruited 31 Chinese patients with SSc and 31 healthy controls matched by age, gender, and body mass index (BMI) from the Second Hospital of Hebei Medical University, between October 2015 and November 2016. Patients met the American College of Rheumatology (ACR)/European League Against Rheumatism (EULAR) 2013 criteria for SSc [18]. Patients were assessed using the mRSS, skin HFU, clinical profiling and serum assessment at enrollment (see below). Disease duration was calculated from onset of the first non-Raynaud’s sign or symptom typical of SSc. Exclusion criteria included (1) overlap with other connective tissue disease as defined by validated criteria; (2) skin thickening due to morphea, eosinophilic fasciitis, scleromyxedema, diabetes mellitus, etc.; (3) infection or ulceration of local skin and widespread edema due to nephrotic syndrome or chronic heart failure; (4) non-provision of voluntary informed consent; and (5) use of corticosteroids or immunosuppressive drugs in the 3 months before admission to the study. Ethics approval was obtained from the ethics committee of the Second Hospital of Hebei Medical University and all participants voluntarily signed a written informed consent conform.

### mRSS

Skin involvement in each patient was semi-quantitatively assessed using the mRSS, by one of the three experienced rheumatologists who were well-trained to be concordant in skin scoring skill and blinded to the HFU results. The mRSS was applied by palpation of 17 skin regions (face, anterior chest, abdomen and bilateral upper arms, forearms, hand dorsa, fingers, thighs, legs and dorsum of the foot ) and was scored on a 0–3 categorical scale (maximum = 51) [8, 19, 20].

### Skin HFU

**Fig. 1:**
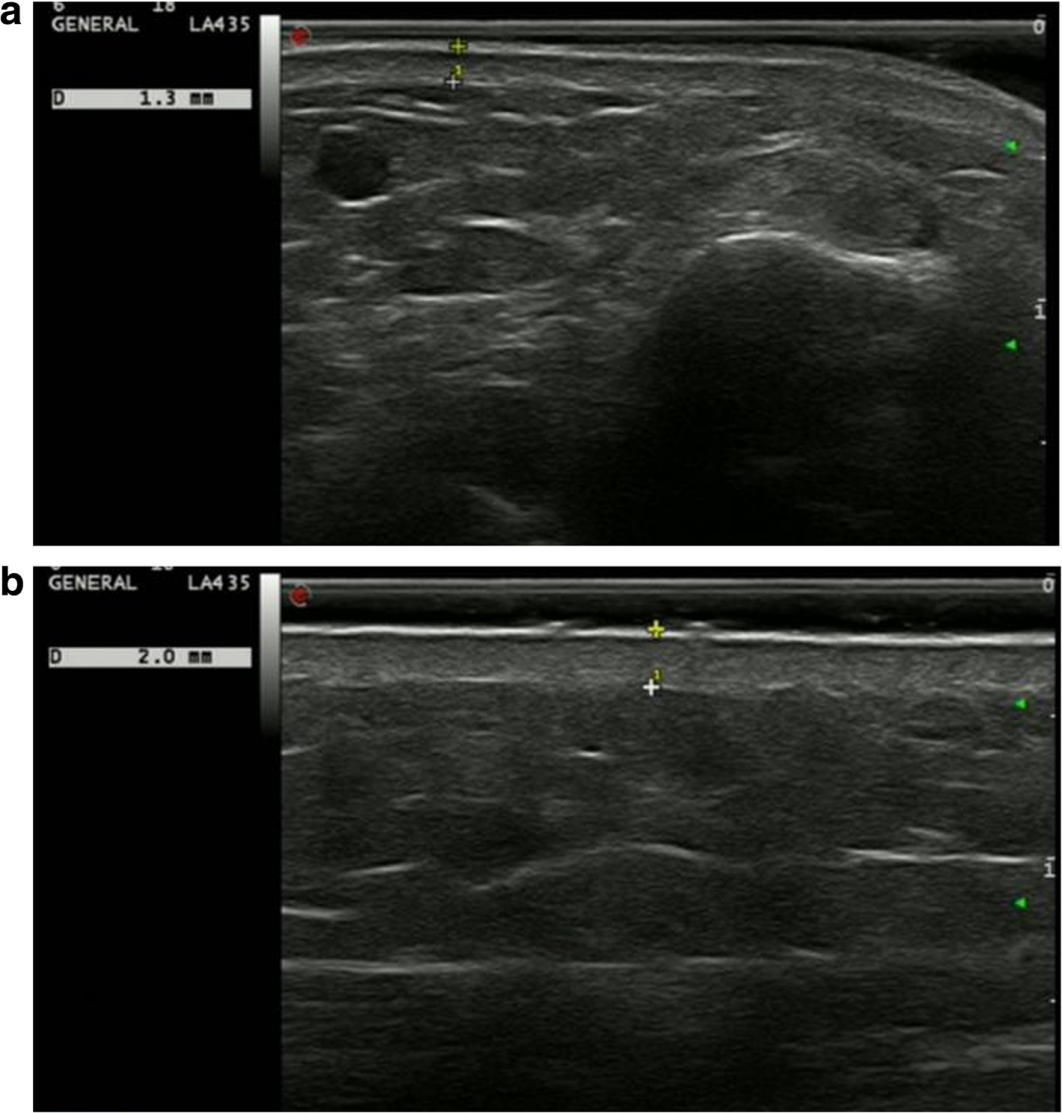
High-frequency ultrasound, 18-MHz echograms (vertical images) highlight the epidermis and dermis at the forearm in a healthy control and a patient with systemic sclerosis. The skin thickness was 1.3 mm (between the plus sign) in the control (**a**), and 2.0 mm (between the plus sign) in the patient (**b**)

Skin thickness including the epidermis and dermis was assessed in both patients and controls by two experienced rheumatologists, who had been engaged in musculoskeletal ultrasound for more than 4 years and were blinded to the clinical data of the subjects. The ultrasound system (ESaote, Italy) was equipped with an 18-MHz probe and all subjects were examined at five anatomical sites—the dorsal skin of the right forearm 3 cm proximal to the wrist, the area between metacarpophalangeal joints II and III of the right hand, the dorsal skin of the proximal phalanx of the right second finger, the skin of the right leg 12 cm proximal to the ankle joint and the sternum 2 cm distal to the upper part of the manubrium [19, 21, 22] (see ultrasound images in Fig. 1). Average values of regional skin thickness and total skin thickness (TST) were measured at three assessments performed horizontally and vertically and were recorded in millimeters, respectively.

### Clinical profiles

Demographic, clinical and laboratory data on patients were systematically collected and the EUSTAR-DAI was calculated according to the EUSTAR 2016 revised standard for disease activity of patients with SSc [23]. The EUSTAR-DAI sums to a maximum weighted 10 points, including six aspects of disease: difference in (Δ) skin mRSS ≥ 18 given a score of 1.5, digital ulcers (DU) given a score of 1.5, tendon friction rubs (TFR) given a score of 2.25, C-reactive protein (CRP) > 1 mg/dL given a score of 2.25 and percentage diffusing capacity of the lung for carbon monoxide (DLCO) < 70% predicted value given a score of 1.0. A EUSTAR-DAI score ≥ 2.5 was defined as active disease [23].

### Statistical analysis

Statistical analyses were conducted using PRISM 5 software. Data were expressed as mean +/− standard deviation or median (interquartile range). Student’s *t* test or the Mann-Whitney U test was performed to compare two groups. Nonparametric testing among three groups was performed using one-way analysis of variance (ANOVA). The Pearson or Spearman methods were used to test correlation. Sensitivity and specificity of skin thickness detection by HFU were evaluated by using receiver operating characteristic (ROC) curve analysis. *P* values <0.05 were considered statistically significant. No correction was made for repeated analyses.

## Results

### Demographics

The mean age of the patients with SSc was 47.23 ± 14.36 years and the average disease duration was 24.0 (32.4) months. Among the 31 patients, 27 were female and 4 were male.

### Skin thickness assessed by HFU

**Fig. 2:**
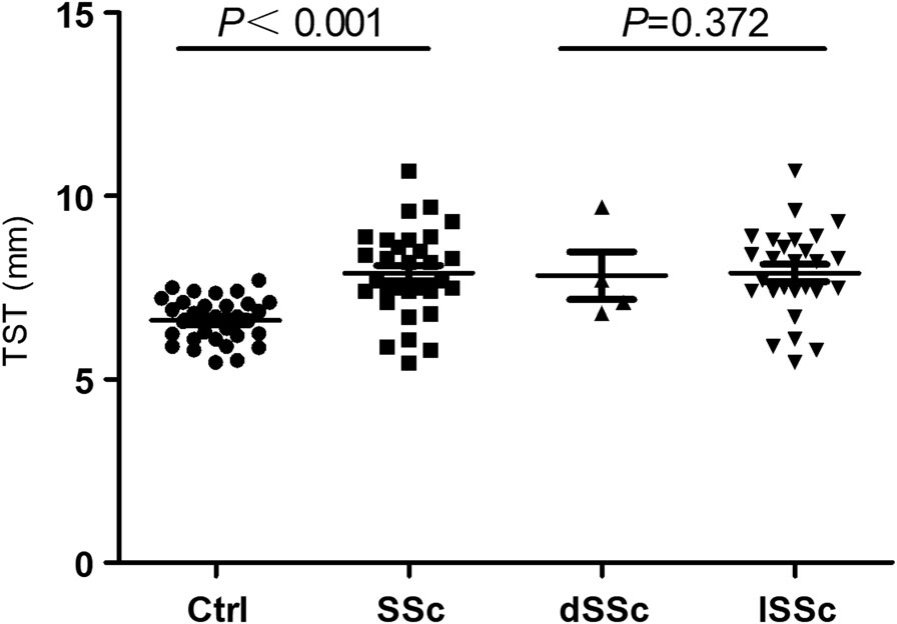
Total skin thickness (TST) evaluated by high-frequency ultrasound in patients with systemic sclerosis (SSc) and healthy controls (Ctrl) (SSc vs Ctrl, *P* < 0.001), and patients with diffuse SSc (dSSc) and patients with limited SSc (lSSc) (dSSc vs lSSc, *P* > 0.05)

**Fig. 3:**
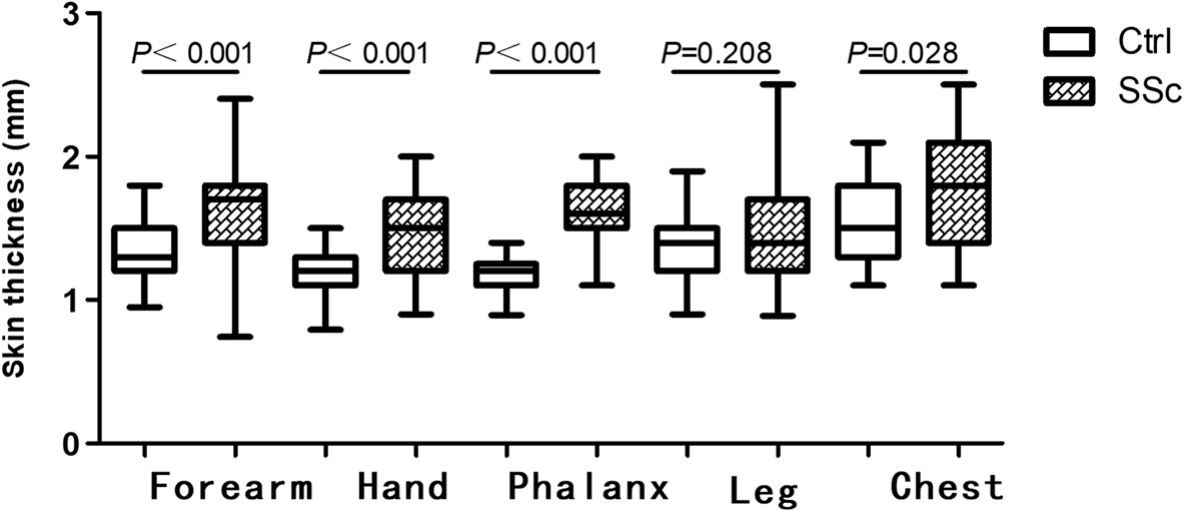
Local skin thickness evaluated by high-frequency ultrasound in patients with systemic sclerosis (SSc) and healthy controls (Ctrl) (SSc vs Ctrl, *P* < 0.05 for all, with the exclusion of the leg). Data are presented for different skin areas, showing the 25th and 75th percentiles of measures as boxes, the median (line within the box) and the minimum and maximum values (whiskers)

We compared total skin thickness (TST) assessed by HFU in the patients with SSc with the total mRSS and found that both demonstrated that the skin was significantly thicker than in healthy controls (*P* < 0.001). However, when measuring localized skin thickness (e.g. on the arm, leg or chest), the skin thickness on the leg was not statistically significant compared to controls (Figs. 2 and 3, Table 1).

**Table 1:** Skin thickness assessed by HFU in patients with SSc and healthy controls

	SSc (skin thickness, mm, *n* = 31)	Ctrl (skin thickness, mm, n = 31)	*P*	Range	
Forearm	1.70(0.40)	1.36 ± 0.19	< 0.001*	0.74–2.40	0.95–1.80
Hand	1.50(0.50)	1.20(0.20)	< 0.001*	0.90–2.00	0.79–1.50
Phalanx	1.60(0.30)	1.20(0.15)	< 0.001*	1.10–2.00	0.90–1.40
Leg	1.40(0.50)	1.40(0.30)	0.208	0.89–2.50	0.90–1.40
Chest	1.80(0.70)	1.50(0.50)	0.028*	1.10–2.50	1.10–1.60
TST	7.89 ± 1.20	6.61 ± 0.61	< 0.001*	5.47–10.70	5.47–7.70

*HFU* high-frequency ultrasound, *SSc* systemic sclerosis, *Ctrl* controls *TST* total skin thickness

*Compared with the healthy controls, *P* < 0.05

Of note, there were also significant differences between patients with limited cutaneous SSc (lSSc) and controls when we divided patients into those with diffuse cutaneous SSc (dSSc, *n* = 4) and those with lSSc (*n* = 27) according to skin involvement. There was no difference in the TST between patients with dSSc and those with lSSc (Fig. 2).

To address skin profiles in patients with different disease duration, the association between ultrasound (US)-defined thickness and disease duration was analyzed. As expected, the TST was negatively correlated with disease duration, with the correlation coefficient indicating moderate to good correlation (*r* = − 0.605, *P* < 0.001) (Additional file 1: Figure S4).

### TST in patients with different mRSS

**Fig. 4:**
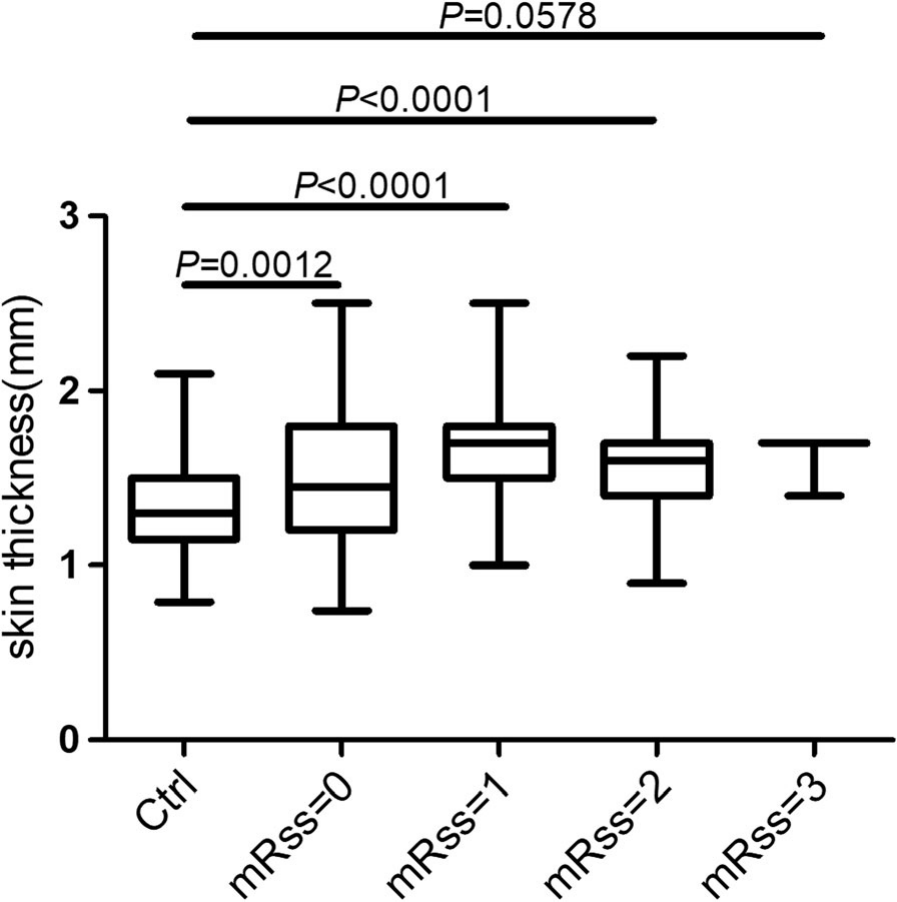
Comparisons of total skin thickness (TST) evaluated by high-frequency ultrasound in healthy control and patients with systemic sclerosis (SSc) with scales of the modified Rodnan skin score (mRSS). Boxes represent the 25th and 75th percentile of measures, and the median (line within the box) and the minimum and maximum values (whiskers) are shown. TST with mRSS = 0, mRSS = 1 and mRSS = 2 in the patients were all significantly higher than in the controls (*P* = 0.0012, *P* <0.0001 and *P* < 0.0001, respectively). No significant difference was found between patients with mRSS = 3 and the controls (*P* = 0.0578)

To detect whether the skin in patients with a clinically normal skin score (mRSS = 0) was thicker than in the healthy controls, we examined the mRSS at five skin sites and local skin thickness assessed by HFU in patients with SSc dichotomously. Of interest, patients with skin thickness score on the mRSS as mRSS = 0, mRSS = 1 and mRSS = 2, all definitely had thicker skin than the controls (*P* < 0.05) (Fig. 4). Although localized skin thickness in the group scored as mRSS = 3 was thicker compared with controls, it was not statistically significant (*P* = 0.057), probably because there were only three skin sites (mRSS = 3) among the total 155 sites.

### Comparing HFU-TST and mRSS in lcSSc

We also compared skin thickness using the full semi-quantitative range of mRSS (0–3+) and HFU in lSSc. Compared with the healthy controls, a skin thickness of mRSS = 0 in patients with SSc assessed by HFU was still thicker than in the normal controls (*P* = 0.001), suggesting skin involvement happened prior to being able to detect it clinically.

### Receiver operating characteristic (ROC) curve analysis for HFU to detect the minimally detectable difference (MDD)

To address the efficiency of TST in SSc, we calculated the sensitivity and specificity for the TST in patients with SSc and the controls using ROC curve analysis. The area under the curve (AUC) was 0.831, standard error was 0.057 (*P* < 0.001), and the 95% confidence intervals (CI) were 0.719 and 0.942 (see Additional file 2: Figure S1). The positive predictive value and negative predictive value were 85.71% and 79.41%, and sensitivity and specificity were 77.40% and 87.10%, respectively. The cutoff value (MDD) for evaluation of thickened skin was 7.4 mm.

The clinical features of the patients with SSc with TST greater than the cutoff from the ROC analysis compared to patients with SSc with TST below the cutoff are shown in Table 2. Patients with greater TST had a higher EUSTAR-DAI, a higher mRSS, higher CRP and lower DLCO (*P* < 0.05) (see also Table 2).

**Table 2:** Clinical profiles of patients with SSc with TST higher and lower than the cutoff in ROC analysis

Feature	Elevated TST (*n* = 24)	Normal TST (*n* = 7)	*P*
Male/female	4/20	0/7	0.247
Age (years)	50.21 ± 14.01	37.00 ± 11.02	0.187
Duration	24.00 (26.40)	56.06 ± 30.17	0.002*
DAI	5.24 ± 2.36	1.84 (1.92)	0.048*
mRSS	10.00 (6.00)	4.00 (5.00)	0.031*
RP	21	7	0.325
ILD	16	1	0.014*
PAH	5	3	0.214
DU	4	2	0.483
Muscular involvement	5	3	0.214
Synovitis	10	2	0.531
Scl-70(+)	10	3	0.955
CRP (mg/L)	8.60 (10.48)	3.52 (4.93)	0.038*
ESR (mm/h)	23.00 (16.25)	48.71 ± 43.19	0.723

*SSc* systemic sclerosis, *TST* total skin thickness, *DAI* European Scleroderma Trial and Research group Disease Activity Index, *RP* Raynaud’s phenomenon, *ILD* interstitial lung disease, *PAH* pulmonary artery hypertension, *DU* digital ulcers, *CRP* C-reactive protein, *ESR* erythrocyte sedimentation rate

*P < 0.05

### Correlation between TST and clinical parameters

There was low-to-moderate, statistically significant, positive correlation between the TST and mRSS (*r* = 0.416, *P* = 0.020) and between the TST and EUSTAR-DAI (*r* = 0.436, *P* = 0.014). The results indicated that to some extent, skin thickness assessed by ultrasound could reflect disease activity. The TST was significantly negatively correlated with disease duration, as we have described before: see Additional file 3: Figure S2, Additional file 4: Figure S3 and Additional file 1: Figure S4.

## Discussion

Skin thickening evaluated by the mRSS is a measurement used often in the clinic to assess patients with either lSSc or dSSc [7, 8, 24]. Unfortunately, relatively large intra-observer or inter-observer variability decreases its applicability for individual patients in the clinic. The coefficient of variation is about 20%, indicating that a change needs to be greater than 20% to be greater than the variability of the measure (similar to the joint counts in rheumatoid arthritis) and is further confounding by edema and the need for a 6–9-month trial [25].

In our study, using an 18-MHz ultrasonic probe, we calculated inter-observer and intra-observer correlation coefficients of 0.88 and 0.93. This corresponds to an intra-observer coefficient of variation of 2.2%, significantly better than that for the mRSS.

Scheja et al. demonstrated in a study of 41 patients with SSc that inter-observer variability when using ultrasound to assess skin thickness in the phalanx, hand and forearm was only 1%, 4.2% and 0.0016%, respectively when using a 20 MHz probe [26]. In addition, Moore et al., who established a 17-point skin HFU scoring system calculated correlation coefficients of 0.93 and 0.95 for inter-observer and intra-observer dermal measurements [14]. These findings support our study results.

ROC analysis defined a minimum detectable difference (MDD) for HFU assessment of skin thickness in SSc in this data set. The AUC was 0.831 (*P* < 0.001) with the best deflection-point at 7.4 mm. Sensitivity was 77.4% and specificity was 87.1% to separate active from inactive disease using the EUSTAR-DAI at a cut point of 2.5. Thus, we identified a skin thickness of 7.4 mm as the MDD that best separated normal skin from skin affected by SSc when we used phalanx/hand/forearm/leg/chest as a composite measure, which were previously defined as the five sites of ultrasound assessment [21, 22].

In view of the facts that more patients with lSSc were recruited into the study, we utilized the composite phalanx/hand/forearm site and local phalanx site separately for extensive ROC analyses (see Additional file 5: Figure S5 and Additional file 6: Figure S6). For skin thickness at the phalanx/hand/forearm sites, the AUC was 0.869 and the cutoff value was 4.3 mm, with the same sensitivity compared as the five sites (77.4%) but with higher specificity (93.5%). For the local phalanx site, the AUC was 0.946 and the cutoff value was 1.3 mm, with 87.1% sensitivity and much more higher specificity (96.8%) to discriminate thickened skin from normal skin. These figures suggest that a better distinction can be shown using fewer ultrasound sites in patients with lSSc, especially if the sites assessed are tailored to the type of patient and to the sites of clinical disease.

In the present study, we demonstrated that TST in patients with SSc is significantly greater than in healthy controls (*P* < 0.001), similar to other research [15, 16]. There were also statistically significantly differences locally, at the forearm, hand, phalanx and chest. There was no difference in skin thickness on the legs in patients with SSc and normal controls. This may have been because in the study there were small numbers of patients recruited with lower extremity involvement, but further research will be needed to examine this finding.

The TST correlated positively with the mRSS and correlated negatively with disease duration, similar to data reported by others [22]. Skin thickness reduced as skin went from interstitial edema through thickening to atrophy [21]. This point could be illustrated by the correlation between the mRSS and TST. The coefficient for correlation between the mRSS and HFU decreased from 0.63 (in patients with disease duration less than 1 year) to 0.40 (in patients with disease duration of 1–3 years) over time in a longitudinal study by Hesselstrant et al. [22]. We found that patients with a normal clinical skin score (mRSS = 0) still had some increased thickening identified by HFU (*P* < 0.01), as have others [27]. This finding may help explain the weaker correlation between the mRSS and HFU over time.

The mRSS is considered by many to be one of the most important aspects in the classification of different SSc subtypes [28]. Sedky et al. showed that ultrasound TST in patients with dSSc was thicker than in those with lSSc (*P* = 0.002), especially in the chest wall [16]. We did not find differences between dSSC and lSSc using HFU-TST. However, the number of patients with dSSc was small (*N* = 4), so the power to identify differences between dSSc and lSSc was very low.

The skin thickness measured by HFU in the chest wall of patients with SSc was greater than in normal controls (*P* < 0.05), despite the fact that 87.1% of our patients had lSSc and thus, by definition, these patients did not have clinical skin thickening on the chest (See Table 2). The meaning of this relative skin thickening at the chest in patients with lSSc as assessed by HFU will require further research in longitudinal studies, although it is congruent with the finding that even when mRSS = 0 in dSSc, the skin is measured as thicker when assessed by HFU.

There was low-to-moderate, positive correlation between HFU-TST and the EUSTAR-DAI (*r* = 0.436, *P* = 0.014) in patients with SSc; this suggests that thickened skin could predict more active disease, although this degree of correlation indicates either the need for many more patients or that active disease is predicted by more than only skin thickness. HFU-TST was also only moderately positively correlated with the mRSS (*r* = 0.416, *P* = 0.020) and the correlation remained in the multivariate regression model (*t* = 0.335, *P* = 0.044). This supports the possibility that the mRSS might reflect the increased skin thickness primarily in the early disease phase and that skin thickness and the mRSS could become disconnected over time [17, 22, 29].

It may be important to recognize and treat SSc in its edematous phase during which patients are more responsive to medication compared with the sclerotic or atrophic phase [29]. Hesselstrand et al. reported that patients in the edematous phase of their disease (usually short duration) had increased skin thickness assessed by HFU, but with low echogenicity (which represents high water content, i.e. more interstitial fluid). It is intriguing in this study that skin thickness negatively correlated with skin echogenicity measured by ultrasound (*P* = 0.001). That relationship implies that the edema results in increased skin thickness and decreased skin echogenicity. Over time, echogenicity increased, correlating with increasing fibrosis [29]. Although our patients had early disease, the finding that TST negatively correlated with disease duration in our data set indicates that these patients had a range of degrees of edema and fibrosis, going from the edematous to the fibrotic/atropic phase as disease duration increased. Overall, the this suggests that objective HFU techniques may be able to differentiate early edematous disease from early fibrosis and may even be able to detect thickening that is not clinically evident. These findings will need corroboration and significant research to ascertain their meaning.

Overall, the mRSS is simple to perform for the assessment of skin changes, it can estimate skin thickness and it samples large areas of the skin, but observer bias and low sensitivity detracts from its accuracy [30]. HFU may be able to examine both skin thickness and interstitial edema, which may mean this modality is able to better examine prognosis and determine drug response [31]. However, standardized imaging, decreasing operator variability, definition of appropriate skin sites and examination of a large range of patients need significant further research [21].

This study has some limitations. First, it is cross-sectional and a longitudinal study would be beneficial, which is being planned. Second, it involves relatively few patients from a single center. However, it is an exploratory effort and has given insights into the MDD and has identified some degree of correlation with a measure of disease activity. A study of more patients is needed, involving a broader set of clinical measures such as the Clinical Response Index for SSc (CRISS) or including gastrointestinal, specific myocardial and lung involvement. Third, we needed to use total skin measurement using HFU because our data precluded separation of the epidermis and dermis. An analysis in a group of patients with earlier more diffuse disease may help address this issue. Further we found no difference between limited and diffuse disease using HFU and the mRSS and did not find differences when assessing skin on the legs and thus, inclusion of more patients with diffuse disease and lower extremity involvement would have been helpful.

## Conclusions

HFU was able to measure skin thickness quantitatively and it correlates with a valid measure of SSc activity, and a minimal detectable difference has been identified. With further research, HFU may be used to effectively and more objectively evaluate skin changes and could be used to predict visceral involvement.

## Additional files

Additional file 1: **Figure S4.** Correlation between the TST and disease duration in patients with SSc. There was negative correlation between the TST and disease duration (*r* = − 0.605, *P* < 0.001). (TIF 3162 kb)

Additional file 2: **Figure S1.** ROC analysis of TST in patients with SSc at the phalanx/hand/forearm/leg/chest sites. The area under the curve (AUC) was 0.831, the cutoff value was 7.4 mm, with sensitivity and specificity of 77.40% and 87.10%, respectively. (TIF 40 kb)

Additional file 3: **Figure S2.** Correlation between TST and DAI in patients with SSc. There was positive correlation between the TST and EUSTAR-DAI (*r* = 0.436, *P* = 0.014). (TIF 3195 kb)

Additional file 4: **Figure S3.** Correlation between the TST and mRSS in patients with SSc. There was positive correlation between the TST and mRSS (*r* = 0.416, *P* = 0.020). (TIF 3144 kb)

Additional file 5: **Figure S5.** ROC analysis of the TST in patients with SSc at the phalanx/hand/forearm sites. The AUC was 0.869 and the cutoff value was 4.3 mm, with 77.4% sensitivity and 93.5% specificity. (TIF 40 kb)

Additional file 6: **Figure S6.** ROC analysis of the TST in patients with SSc at the phalanx site. The AUC was 0.946 and the cutoff value was 1.3 mm, with 87.1% sensitivity and much higher specificity (96.8%). (TIF 39 kb)

## Abbreviations

ACR: American College of Rheumatology
ANOVA: One-way analysis of variance
AUC: Area under the curve
BMI: Body mass index
CRISS: Clinical Response Index for SSc
CRP: C-reactive protein
DLCO: Diffusing capacity of the lung for carbon monoxide
dSSc: Diffuse cutaneous systemic sclerosis
DU: Digital ulcers
EULAR: European League Against Rheumatism
EUSTAR-DAI: European Scleroderma Trial and Research-Disease Activity Index
HFU: High-frequency ultrasound
lSSc: Limited cutaneous SSc
MDD: Minimally detectable difference
mRSS: Modified Rodnan skin score
ROC: Receiver operating characteristic
SSc: Systemic sclerosis
TFR: Tendon friction rubs
TFR: Tendon friction rubs
TST: Total skin thickness
