# Supplementary figures and images for: High-frequency ultrasound of the skin in systemic sclerosis: an exploratory study to examine correlation with disease activity and to define the minimally detectable difference
Source: Arthritis Res Ther. 2018 Aug 16;20:181. doi: 10.1186/s13075-018-1686-9 (PMC6097225; doi:10.1186/s13075-018-1686-9)

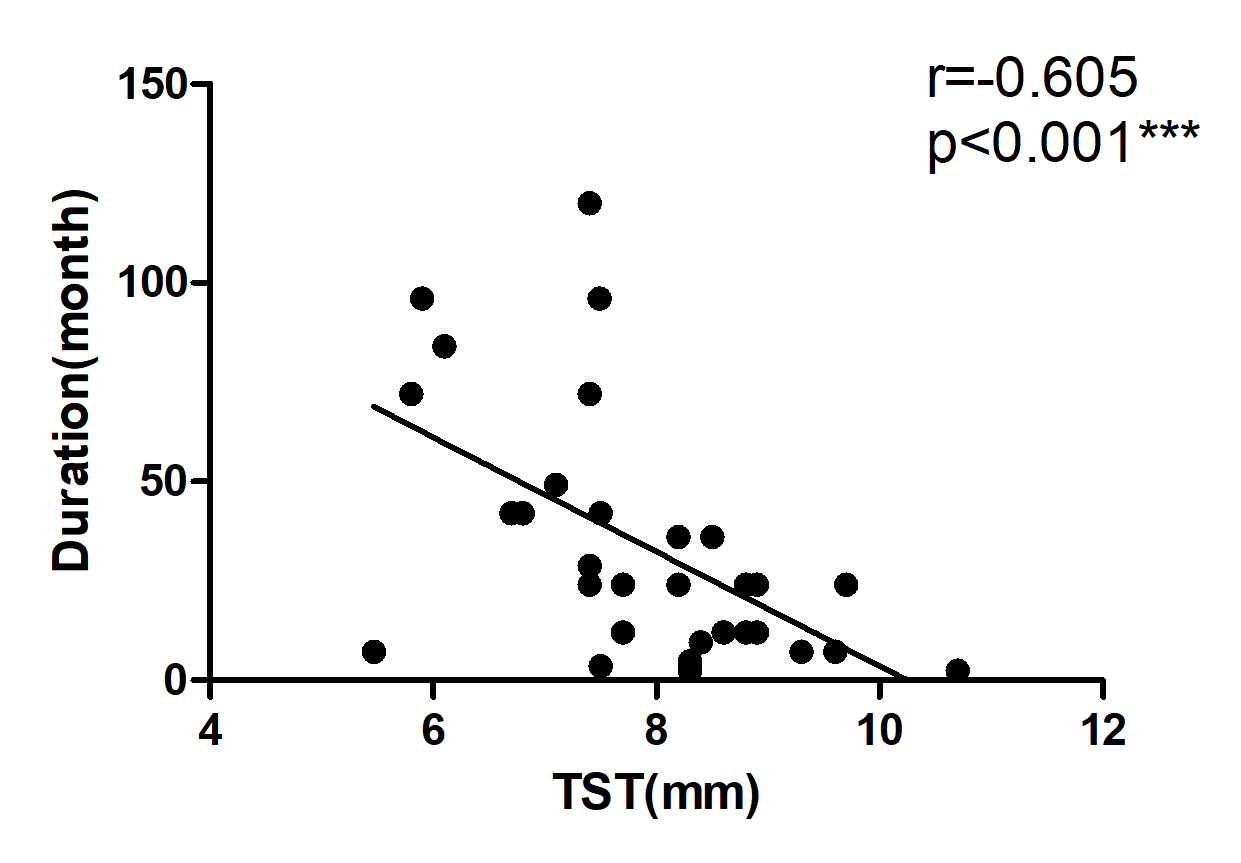

Supplement: Supplementary file 1 — Figure S4. Correlation between the TST and disease duration in patients with SSc. There was negative correlation between the TST and disease duration (r = − 0.605, P < 0.001). (TIF 3162 kb) [file 13075_2018_1686_MOESM1_ESM.tif]

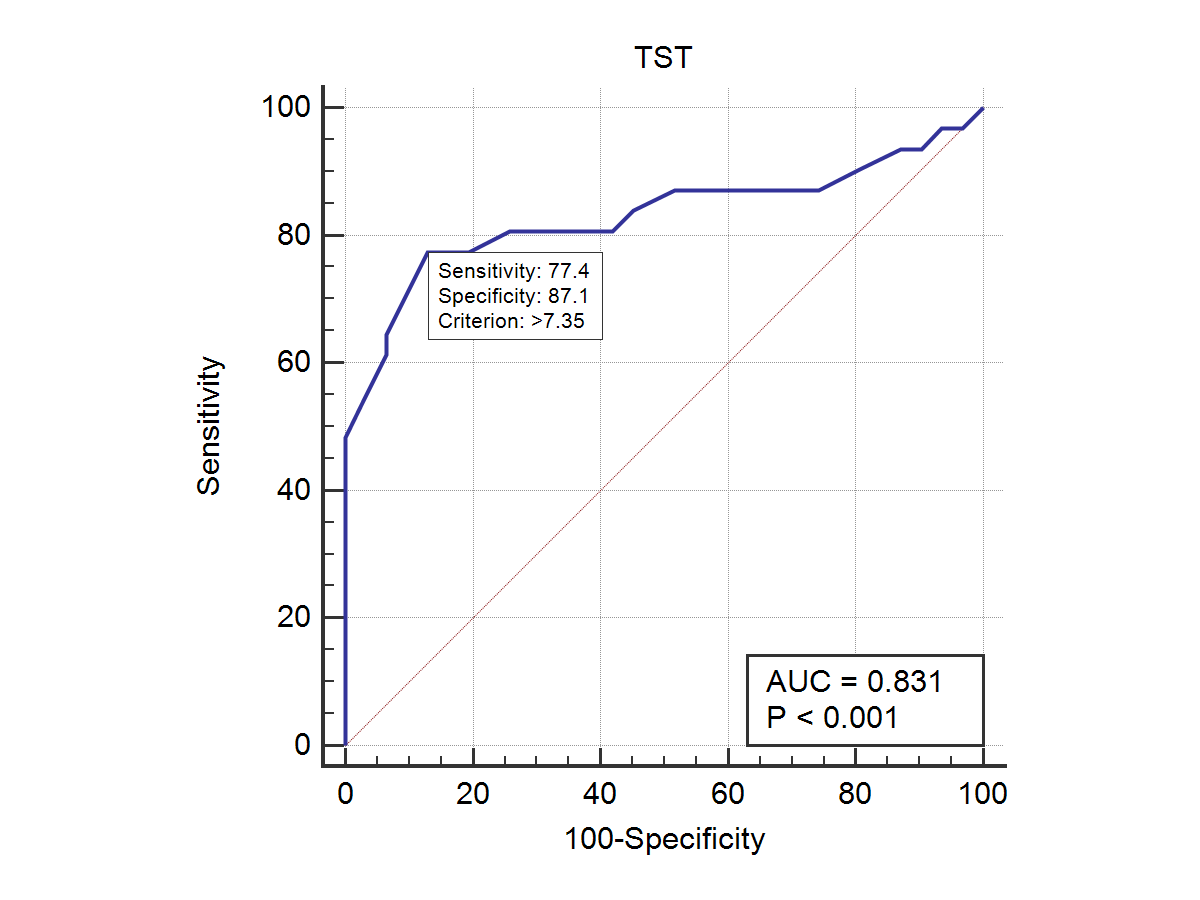

Supplement: Supplementary file 2 — Figure S1. ROC analysis of TST in patients with SSc at the phalanx/hand/forearm/leg/chest sites. The area under the curve (AUC) was 0.831, the cutoff value was 7.4 mm, with sensitivity and specificity of 77.40% and 87.10%, respectively. (TIF 40 kb) [file 13075_2018_1686_MOESM2_ESM.tif]

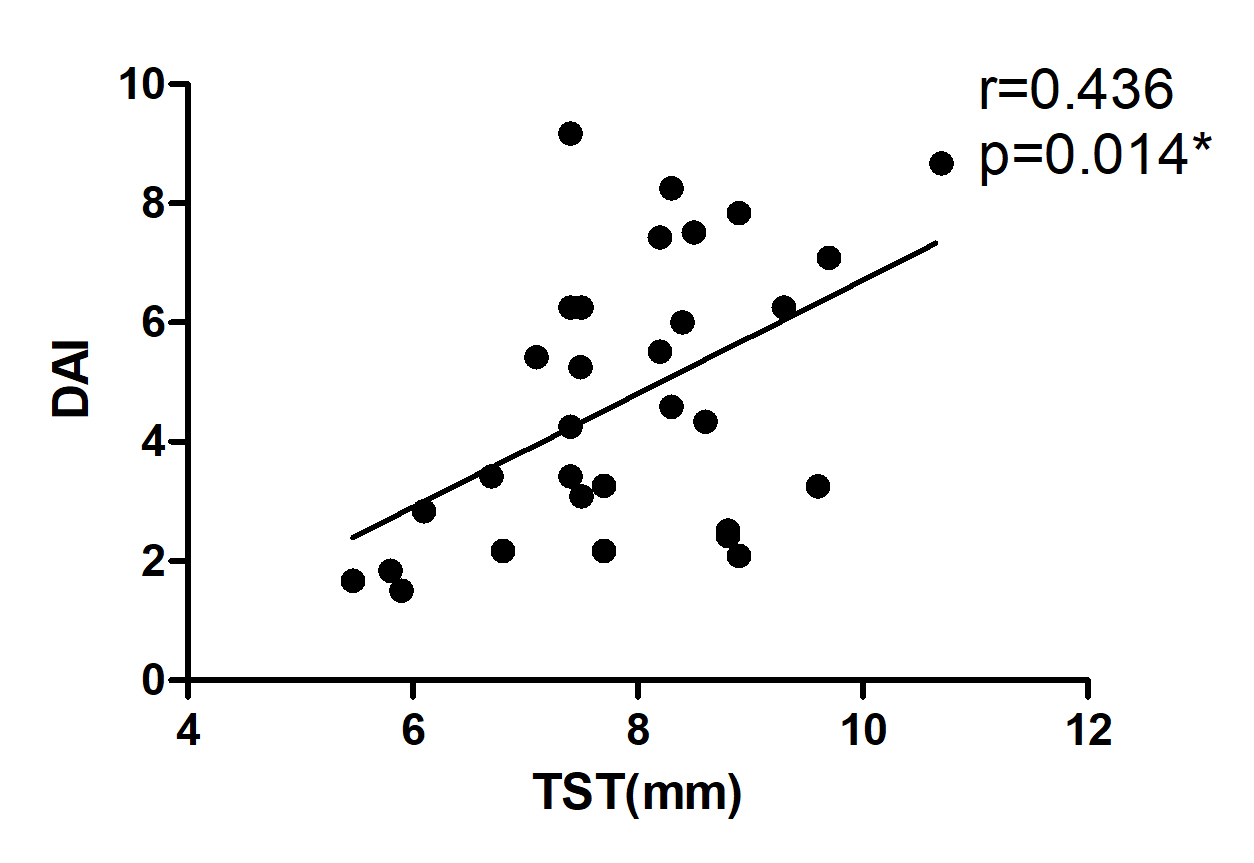

Supplement: Supplementary file 3 — Figure S2. Correlation between TST and DAI in patients with SSc. There was positive correlation between the TST and EUSTAR-DAI (r = 0.436, P = 0.014). (TIF 3195 kb) [file 13075_2018_1686_MOESM3_ESM.tif]

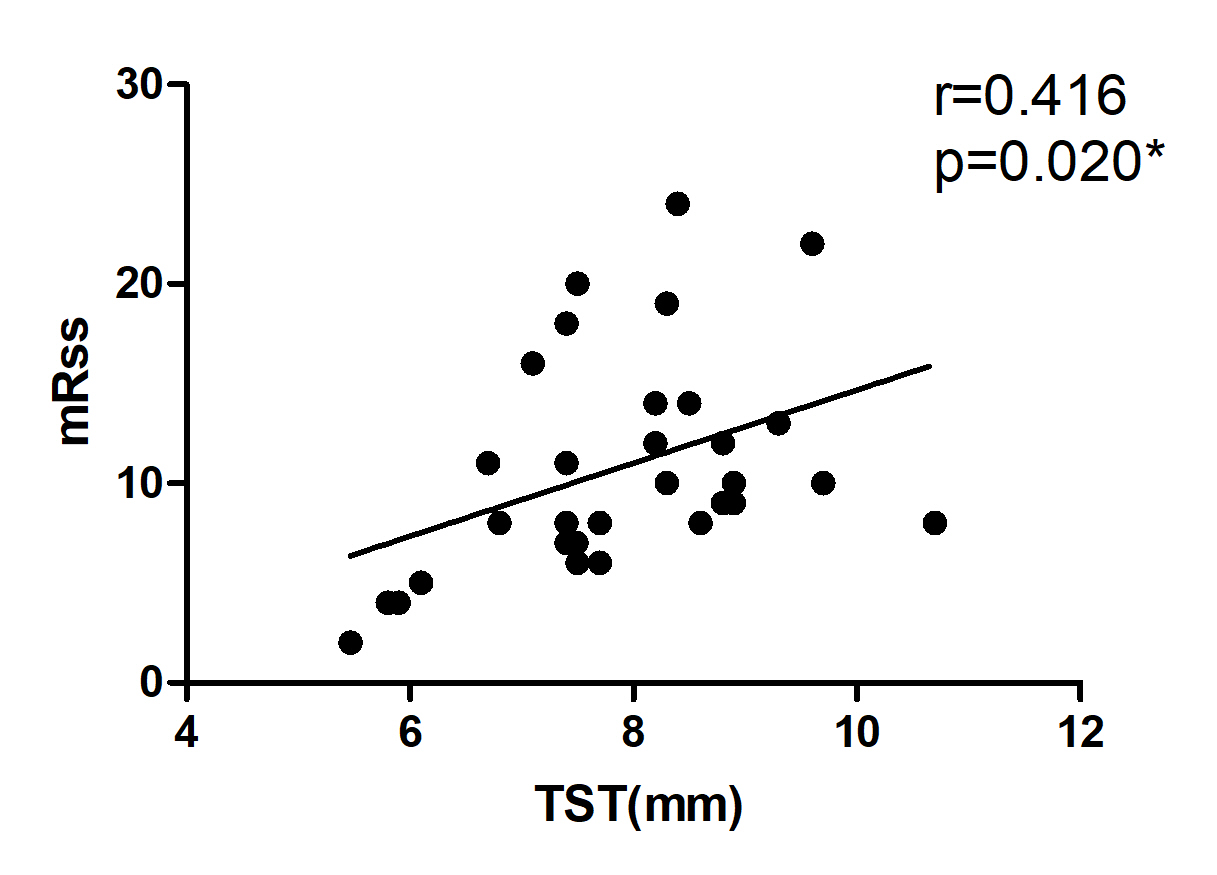

Supplement: Supplementary file 4 — Figure S3. Correlation between the TST and mRSS in patients with SSc. There was positive correlation between the TST and mRSS (r = 0.416, P = 0.020). (TIF 3144 kb) [file 13075_2018_1686_MOESM4_ESM.tif]

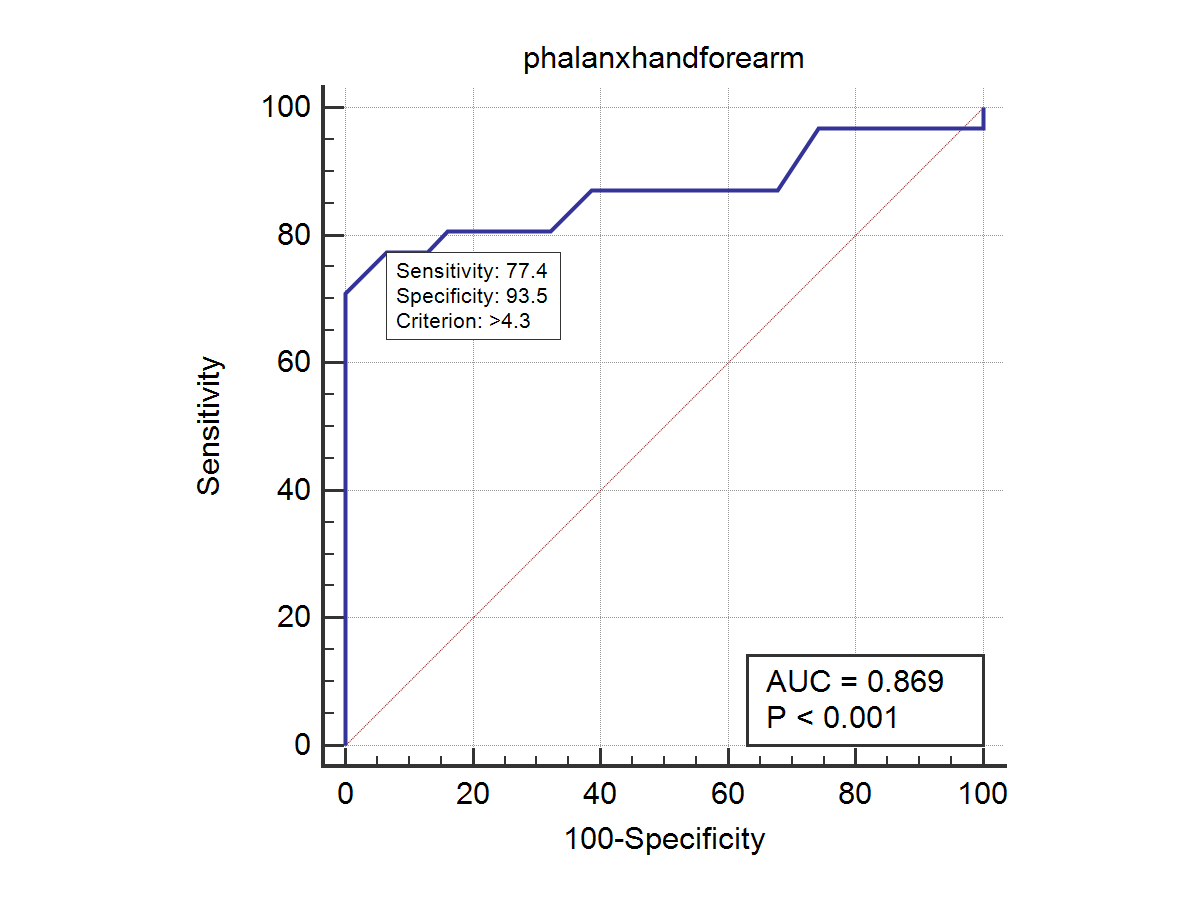

Supplement: Supplementary file 5 — Figure S5. ROC analysis of the TST in patients with SSc at the phalanx/hand/forearm sites. The AUC was 0.869 and the cutoff value was 4.3 mm, with 77.4% sensitivity and 93.5% specificity. (TIF 40 kb) [file 13075_2018_1686_MOESM5_ESM.tif]

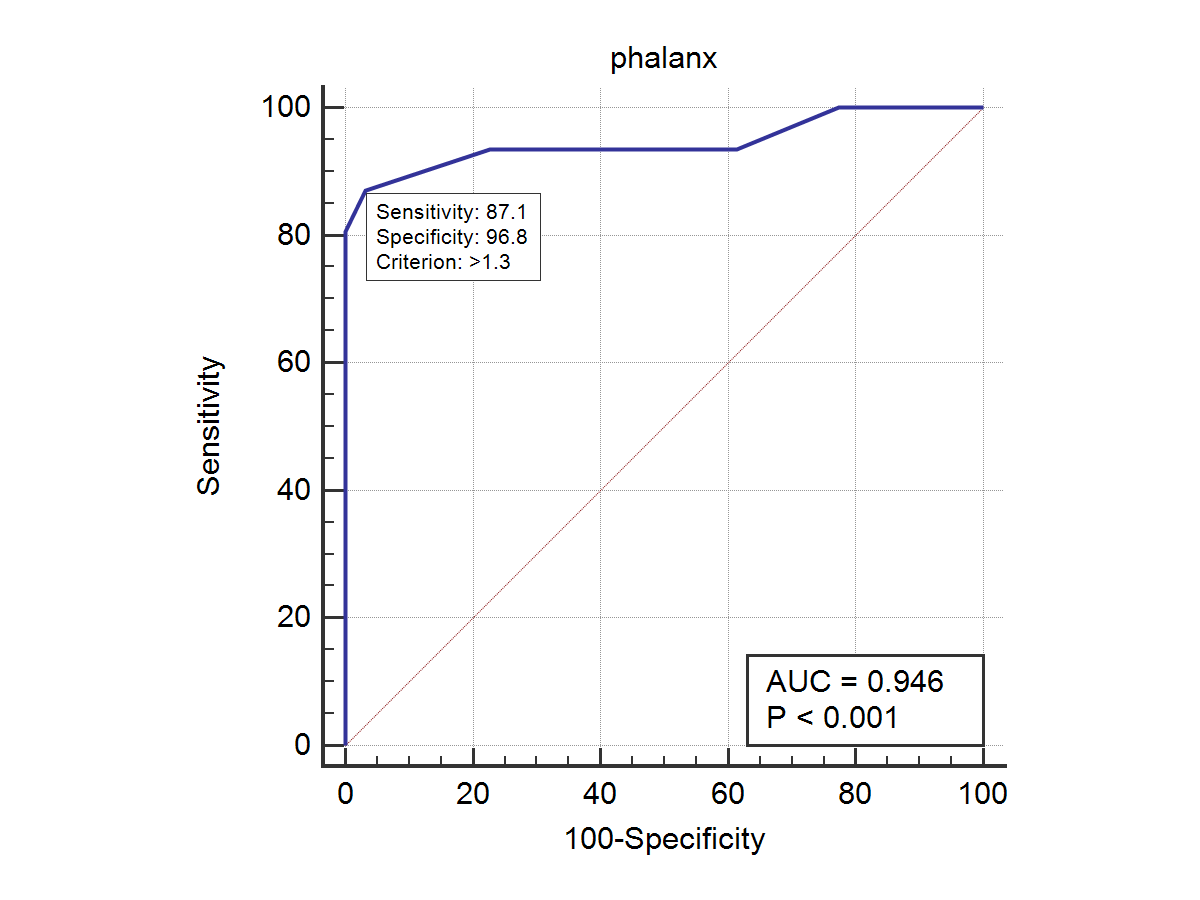

Supplement: Supplementary file 6 — Figure S6. ROC analysis of the TST in patients with SSc at the phalanx site. The AUC was 0.946 and the cutoff value was 1.3 mm, with 87.1% sensitivity and much higher specificity (96.8%). (TIF 39 kb) [file 13075_2018_1686_MOESM6_ESM.tif]
